# Multiplex gene regulation by CRISPR-ddCpf1

**DOI:** 10.1038/celldisc.2017.18

**Published:** 2017-06-06

**Authors:** Xiaochun Zhang, Jingman Wang, Qiuxiang Cheng, Xuan Zheng, Guoping Zhao, Jin Wang

**Affiliations:** 1 Key Laboratory of Synthetic Biology, Institute of Plant Physiology and Ecology, Shanghai Institutes for Biological Sciences, Chinese Academy of Sciences, Shanghai, China; 2 School of Life Science and Technology, Shanghai Tech University, Shanghai, China; 3 University of Chinese Academy of Sciences, Beijing, China; 4 State Engineering Laboratory of Medical Key Technologies Application of Synthetic Biology, Shenzhen Second People’s Hospital, The First Affiliated Hospital of Shenzhen University, Shenzhen, China; 5 Sun Yat-sen University Cancer Center, Guangzhou, China; 6 Shanghai Tolo Biotechnology Company Limited, Shanghai, China

**Keywords:** CRISPR, Cpf1, DNase-dead Cpf1 (ddCpf1), CRISPRi, multiplex gene regulation

## Abstract

The clustered regularly interspaced short palindromic repeats (CRISPR)/dCas9 system has been widely applied in both transcriptional regulation and epigenetic studies. However, for multiple targets, independent expression of multiple single guide RNAs (sgRNAs) is needed, which is less convenient. To address the problem, we employed a DNase-dead Cpf1 mutant (ddCpf1) for multiplex gene regulation. We demonstrated that ddCpf1 alone could be employed for gene repression in *Escherichia coli*, and the repression was more effective with CRISPR RNAs (crRNAs) specifically targeting to the template strand of its target genes, which was different from that of dCas9. When targeting the promoter region, both strands showed effective repression by the ddCpf1/crRNA complex. The whole-transcriptome RNA-seq technique was further employed to demonstrate the high specificity of ddCpf1-mediated repression. Besides, we proved that the remaining RNase activity in ddCpf1 was capable of processing a precursor CRISPR array to simply generate multiple mature crRNAs *in vivo*, facilitating multiplex gene regulation. With the employment of this multiplex gene regulation strategy, we also showed how to quickly screen a library of candidate targets, that is, the two-component systems in *E. coli*. Therefore, based on our findings here, the CRISPR-ddCpf1 system may be further developed and widely applied in both biological research and clinical studies.

## Introduction

The clustered regularly interspaced short palindromic repeats (CRISPR) adaptive immune systems are widely distributed among almost all archaea and a large number of bacteria, protecting microbes against invasion by foreign DNAs, such as viruses [1]. The CRISPR-associated protein Cas9, which belongs to class 2 type II CRISPR-Cas system, has been extensively developed as a powerful tool for genome editing in both prokaryotes and eukaryotes [2,3,4,5]. With catalytically dead Cas9 (dCas9), the CRISPR/dCas9 system can be repurposed for targeting genomic DNA without introducing a double-stranded break [6]. dCas9 was first demonstrated for gene regulation in *Escherichia coli*, and the technology was named as CRIPSR interference (CRISPRi). A CRISPRi system consists of dCas9 and a single guide RNA (sgRNA) and the guide sequences in the sgRNA are responsible for specific recognition of target gene. As CRISPRi is of much convenience and high efficiency, it has been widely applied for efficient gene regulation [7,8,9,10,11,12,13,14,15] and epigenetic studies [16,17,18,19,] in both prokaryotes and eukaryotes.

In most cases, CRISPRi is designed for one target, which can be achieved by coexpression of a single sgRNA and dCas9. However, for multiplex gene regulation or epigenetic modifications, multiple sgRNAs may need to be independently expressed [20], and the construction procedure is time-consuming. As the studied gene networks become more and more complicated, it would be very useful to develop a convenient multiplex targeting system.

Besides of Cas9, another CRISPR-Cas protein Cpf1, which belongs to the class 2 type V-A CRISPR-Cas system, is also widely applied for genome editing in many organisms [21,22,23,24,25,26, 27,28]. Similar to Cas9, Cpf1 also cleaves double-stranded DNA and introduces double-stranded breaks at the recognition site. However, unlike Cas9, only the crRNA is required by Cpf1. Besides, Cpf1 also possesses the RNase activity and processes its own precursor crRNA [29]. Therefore, Cpf1 is so far the most minimalistic CRISPR-Cas systems with dual DNase and RNase activities [29]. Recently, its dual activities have been employed to process a single customized CRISPR array with its RNase activity and then cut target DNAs with its DNase activity, allowing for multiplex genome editing in both mammalian cells and rice [30, 31]. Because multiple mature crRNA can be conveniently obtained, the system can thus be applied for convenient multiplex genome editing.

Different from Cas9, which contains the RuvC and HNH domains for cleavage of the non-target strand and target strand, respectively [32], Cpf1 lacks HNH domain but contains a newly found Nuc domain [33, 34]. As mutation of the RvuC domain would result in the loss of cleavage activity against both strands of target DNA, the RuvC-mutated *Francisella tularensis* Cpf1 (FnCpf1) has previously been employed for protospacer-adjacent motif (PAM)-screen achieved by NOT-gate repression (SCANR) in *E. coli *[35], indicating the possibility of employing Cpf1 in gene regulation. As distinct domains have been characterized for the DNase and the RNase activities, inactivation of the DNase activity has no influence on its RNase activity [29]. Therefore, the DNase-dead Cpf1 (namely ddCpf1) in theory can be employed to process its precursor crRNA as well as a customized CRISPR array. And in this study, we employed the ddCpf1, which remained the RNase activity, to process a precursor CRISPR array, simply generating multiple mature crRNAs for convenient multiplex gene regulation.

## Results

### ddCpf1 can effectively repress both transcription elongation and initiation of target genes

**Figure 1:**
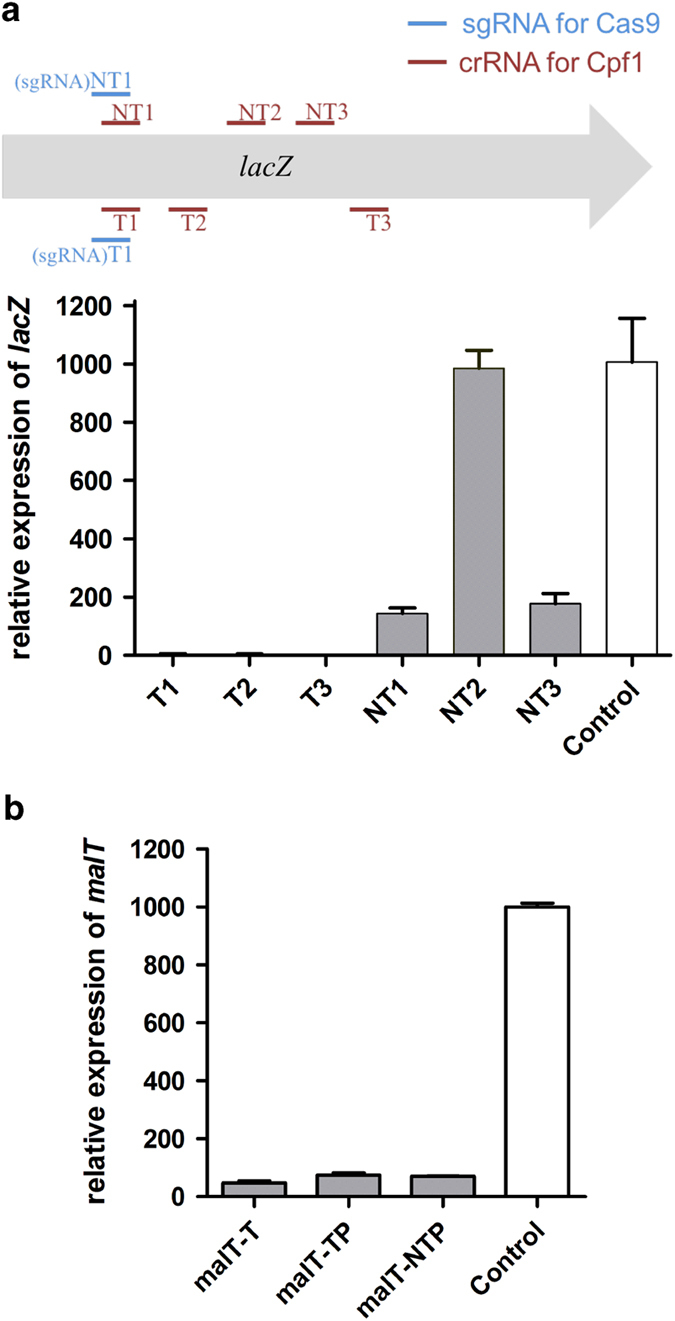
Programmable gene repression by ddCpf1/crRNA. (**a**) ddCpf1-mediated repression of the transcription *lacZ* in MG1655. Positions of the crRNAs and sgRNAs designed for *lacZ* were illustrated, targeting to either the T strand or the NT strand of *lacZ*. Although only the crRNAs targeting to the T strand showed remarkable repression. The transcription of *lacZ* in cells expressing ddCpf1 alone was employed as a control, and the value was normalized to 1000. Two sgRNAs were also designed, and the dCas9-mediated repression was more effective with sgRNA targeting to the NT strand, which could be found in Supplementary Figure S1. (**b**) ddCpf1-mediated repression of the transcription *malT* in MG1655. crRNAs were designed to target both the promoter region and the T strand in the coding region of *malT*, and the guide sequences could be found in Supplementary Table S1. The transcription of *malT* in cells expressing ddCpf1 only was employed as a control, and the value was normalized to 1 000. Symbols of malT-T, malT-TP and malT-NTP represented crRNAs targeting to the T strand in the coding region, T strand in the promoter region and the NT strand in the promoter region of *malT* gene, respectively.

The E993A mutation in the RuvC domain of *Acidaminococcus sp.* Cpf1 (AsCpf1) generates a ddCpf1, which would lose the DNase activities against both strands of target DNA [34]. To verify whether ddCpf1 could efficiently and selectively repress the transcription of its target genes, we coexpressed ddCpf1 and crRNAs in *E. coli*. The expression of ddCpf1 was driven by a constitutive BioBrick promoter of J23100 in a plasmid containing p15A ori, while the crRNA transcription was driven by the BioBrick promoter of J23109 in a colE1 plasmid. We designed three crRNAs targeting to the template DNA strand (T strand) and three targeting to the non-template DNA strand (NT strand) in the coding region of *lacZ*. Isopropylthio-β-galactoside (IPTG) was added to relieve the LacI repression and induce the transcription of *lac* operon. Our results showed that all crRNAs targeting to the T strand showed effective gene silencing (~330-fold repression for all three sites), whereas those targeting to the NT strand were either less effective (~6-fold repression for NT1 and NT3 sites) or ineffective at all (for example, for NT2 site; Figure 1a). To exclude the possible regulatory role of crRNA, we expressed T1 alone and found the transcription of *lacZ* was not significantly changed (Supplementary Figure S1). Besides, when a crRNA (crRNA-T4) targeting to a sequence without a proper PAM in the T strand, there was also no significant repression effect of *lacZ* expression (Supplementary Figure S1). Therefore, the above results demonstrated the repression of *lacZ* transcription was conducted by the ddCpf1/crRNA complex, and a proper PAM sequence was necessary for the repression.

Considering dCas9/sgRNA can specifically block the transcriptional initiation through binding to the promoter regions of its target genes, we then tested whether the ddCpf1/crRNA complex had similar regulatory function. We designed a pair of crRNAs, targeting to the NT and T strands in the promoter region of *malT*, respectively, and found both crRNAs led to effective silencing effect (that is, ~14 folds for both; Figure 1b). The results therefore demonstrated that ddCpf1-mediated repression of transcriptional initiation was independent of the targeting strand, which was similar to that of dCas9 [6].

### The ddCpf1/crRNA complex may function as a road block to prevent transcription elongation

**Figure 2:**
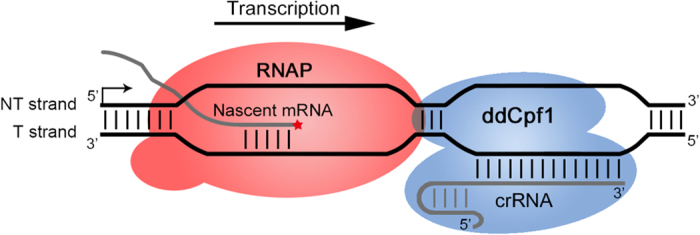
A proposed model for ddCpf1-mediated repression of gene transcription. The ddCpf1/crRNA complex bound to the T strand in the coding sequence of a target gene, blocking the transcription elongation of the RNAP. Alternatively, the ddCpf1/crRNA complex could bind to the promoter region of its target gene to block the transcription initiation, which was not shown in this model.

Interestingly, the dCas9/sgRNA complex effectively blocks the transcription elongation through targeting to the NT strand in both previous findings [6] and in this study (Supplementary Figure S2). When binding to the NT strand, the dCas9/sgRNA complex may physically block the transcriptional elongation; otherwise, the sgRNA could be unzipped by the helicase activity of the RNA polymerase (RNAP), resulting in the loss of repressive activity of the dCas9/sgRNA complex [6]. This hypothesis is also suitable for the ddCpf1/crRNA complex, as only when the complex targeting to the T strand the crRNA can escape from the helicase activity of RNAP and lead to physical collision between the complex and the elongating RNAP (Figure 2), therefore blocking the RNAP elongation and repressing the target gene’s transcription.

When the ddCpf1/crRNA complex targets to the promoter region, there is no strand bias (Figure 1b). Probably, the complex functions as a stereospecific blockade to compete with RNAP for binding to the promoter, which will prevent the transcription initiation in a similar way to that of dCas9.

### ddCpf1-mediated repression is of high specificity

**Figure 3:**
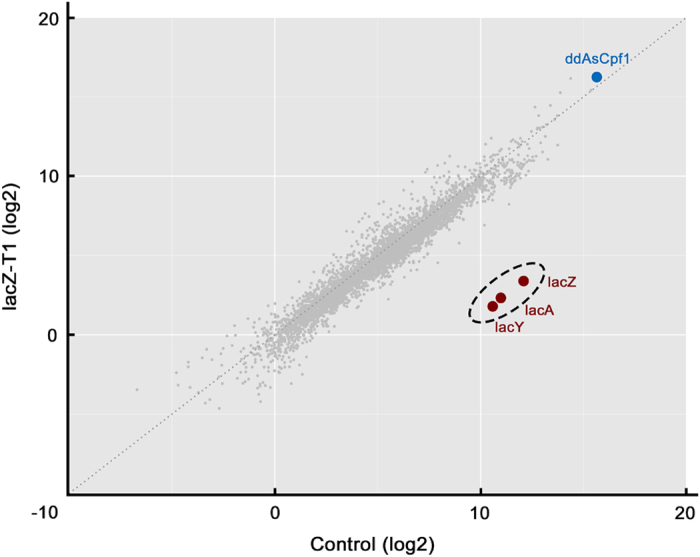
Whole-transcriptome RNA-seq analysis of the specificity of ddCpf1-mediated repression. Cells expressing ddCpf1 with or without lacZ-T1 crRNA were analyzed, and according to the FPKM values, only the transcription of the *lac* operon was remarkably repressed, demonstrating the high specificity of ddCpf1-mediated repression. Genes of *ddAsCpf1*, *lacZ*, *lacY* and *LacA* were highlighted.

To evaluate the targeting specificity of ddCpf1-mediated repression, we employed the whole-transcriptome RNA-seq to analyze the regulatory profiles of cells expressing ddCpf1 with and without a crRNA. When the *lacZ*-crRNA(T1) targeting to the T1 site in *lacZ* was coexpressed with ddCpf1, only the *lac* operon was remarkably repressed, while no other genes showed significant changes in expression (Figure 3). Therefore, based on the RNA-seq results, one could conclude that the ddCpf1-mediated gene repression was of high specificity and showed no significant off-target effects.

### ddCpf1 can be employed for efficient multiplex gene repression

As the wild-type Cpf1 has been demonstrated to process a customized crRNA array both *in vitro* and *in vivo *[30], we next tested whether ddCpf1 still remained this activity. We chose four genes (*malT*, *proP*, *degP* and *rseA*) and constructed a crRNA array expressing pre-crRNAs in the order of *malT-proP-degP-rseA* (array 1), using 19-nt direct repeat (DR) and 23-nt guide because this combination has been recently demonstrated to have good performance [30].

**Figure 4:**
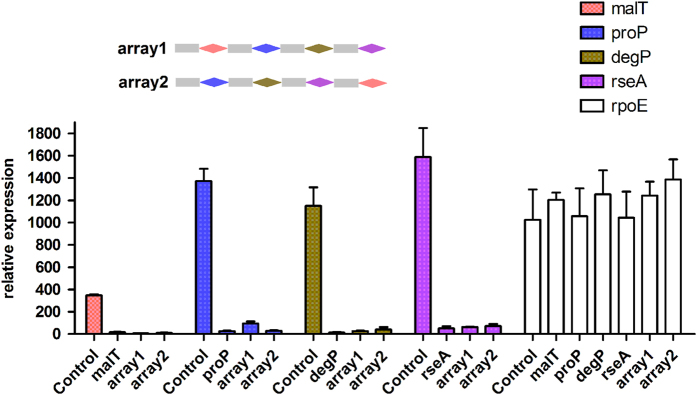
Multiplex gene repression with ddCpf1 and a crRNA array. Four target genes (*malT*, *proP*, *degP* and *rseA*) were analyzed, employing the non-target *rpoE* gene as an internal control. The order of the target genes was shown, which differed in array 1 from array 2. The transcriptional level of each gene was analyzed in cells expressing ddCpf1 with either individual crRNA or crRNA arrays, and cells expressing ddCpf1 only were employed as a control. For *rpoE* gene, its transcriptional level was analyzed in cells individually expressing all the tested crRNAs, and its value in cells expressing ddCpf1 only was normalized to 1000.

When array 1 was coexpressed with ddCpf1, all four genes were repressed with similar folds to those expressing ddCpf1 and individual crRNAs, while the transcription of the non-targeted *rpoE* gene showed no significant change (Figure 4). Similarly, coexpression of ddCpf1 and array 2, which was constructed in the order of *proP-degP-rseA-malT*, also resulted in effective silencing of all targeted genes (Figure 4). In the above two arrays, the crRNA positions differed in each array but the transcriptional repression of target genes was not significantly affected, which therefore indicated that the crRNA order did not influence the repression efficiency. Notably, similar results were also observed in the Cpf1-mediated multiplex gene editing [30, 31].

### The multiplex silencing strategy allows for prompt screening of candidate targets

The practicability of multiplex gene repression allows for convenient repression of multiple genes by one construct, which could be employed for prompt screening of a library of candidates. To demonstrate this potential application, we used this multigene silencing strategy to quickly characterize the two-component systems (TCS) in *E. coli*. A typical TCS is comprised of a histidine kinase and a response regulator (RR), where histidine kinase senses extracellular stimuli and RR regulates target genes’ expression to help bacteria cope with the environmental stresses [36]. In *E. coli*, there are at least 30 different TCSs, including 30 histidine kinases and 32 RRs [37].

**Figure 5:**
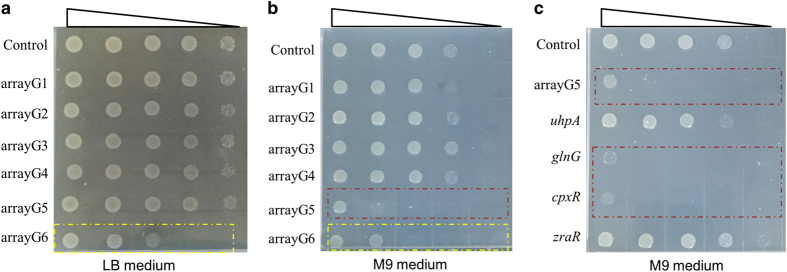
Prompt characterization of TCSs in *E. coli* with the ddCpf1-mediated multiplex gene repression strategy. The 32 RRs in *E. coli* were divided into 6 groups (Supplementary Tables S3 and S4), and the growth phenotypes were analyzed by culture in either rich LB medium (**a**) or minimal M9 medium (**b**). In **c**, cells expressing ddCpf1 and individual crRNAs that targeted genes in arrayG5 were cultured in M9 medium, employing arrayG5 as a control. The growth-deficient strains, which expressed ddCpf1 and arrayG5 (or crRNAs targeting genes in arrayG5), were marked with the red dashed box, while the yellow dashed box marked the slow growth phenotype of arrayG6. A gradient dilution of cells with sterile water was performed, and different amounts of cells (that is, 4.0×10^5^, 4.0×10^4^, 4.0×10^3^, 4.0×10^2^ and 4.0×10^1^) were dotted on plates. Cells expressing ddCpf1 only were employed as the positive control.

To mimic the quick characterization of the TCSs, we divided the 32 RRs into 6 groups and accordingly constructed 6 crRNA arrays (that is, arrayG1 to arrayG6), which were then individually coexpressed with ddCpf1 to repress the transcription of genes in each group (Supplementary Tables S3 and S4). When grown on M9 minimal medium, cells expressing arrayG5 and ddCpf1 showed obvious growth defect (Figure 5b). Because the growth could be recovered by glutamine supplementation (Supplementary Figure S3A), the growth defect was probably caused by deficiency in nitrogen assimilation, indicating that the genes in group 5 were involved in the process of nitrogen assimilation. We next individually silenced the four genes in group 5 by coexpression of ddCpf1 and individual crRNA (Supplementary Figure S4), and found that both TCSs of *glnL/glnG* and *cpxA/cpxR* were related to the growth defect on M9 plate (Figure 5c). As GlnLG regulated at least 20 genes involved in nitrogen metabolism [38], it was easy to understand the growth defect caused by the repression of *glnLG* expression. While for CpxRA, mutation of which was known to cause hypersensitiveness to several amino acids at an alkaline pH and to aminoglycosides [38], we here found the system might also be involved in the regulation of ammonium assimilation, and the detailed mechanism could be subject to further investigation. Moreover, through screening the crRNA arrays, we also found the repression of *arcAB* expression lead to impaired cell growth even in both rich lysogeny broth (LB) medium and M9 medium (Figure 5a and b, Supplementary Figure S3B), which was consistent with a previous finding [38]. Therefore, with the availability of this multiplex silencing strategy, the process of screening of a large number of candidates can surely be accelerated.

## Discussion

It is well known that many important cellular activities require co-ordinated transcription of multiple genes. Therefore, the ability to precisely regulate multiple genes’ transcription may greatly promote the understanding of these complicated gene networks. Although traditional techniques such as RNA-mediated interference (RNAi) using either small interfering RNAs or short hairpin RNAs can be employed for gene suppression in eukaryotes [39], the situation of lacking efficient and convenient tools for gene regulation in prokaryotes lasted till the development of CRISPRi with dCas9. Besides, the potential off-target effect of RNAi in eukaryotes is also a matter of concern [40]. Instead, CRISPRi has been well demonstrated for its convenience and high specificity, and has been widely adopted for programmable gene repression in both prokaryotes and eukaryotes. Besides of CRISPRi, CRISPR/dCas9 system has also been developed for other applications, such as gene-specific activation (CRISPRa) and epigenetic studies.

The only inconvenience of the CRISPR/dCas9 system may exist in the targeting of multiple genes, when multiple sgRNA expression cassettes must be constructed. To address this problem, we here developed the ddCpf1-mediated CRISPRi system. Similar to dCas9, the ddCpf1-mediated target-specific repression was also of high specificity, which was consistent with previous findings that genome editing with the wild-type Cpf1 was of extremely low off-target effect [41, 42]. Moreover, with the utility of CRISPR array, ddCpf1 was demonstrated as an efficient tool for convenient repression of multiple genes in *E. coli*.

During the experiments, we also found that the transformation efficiency of ddCpf1 plasmids was obviously lower than dCas9 and those without ddCpf1 (data not shown). Although successful *E. coli* transformants could always be obtained for subsequent transcriptional analyses, the in-depth mechanisms might be an interesting question subject to further investigation.

Besides of *E. coli*, we also tested the efficiency of gene repression by ddCpf1 alone in human HEK293T cells, that is, through directly targeting different positions of *DNMT1*, including the T strand, NT strand and the promoter region (Supplementary Figure S5). However, no significant repression was observed for all tested crRNAs, which was similar to those observed for dCas9 alone [6], where only two out of seven designed sgRNAs showed moderate repression. As fusion of dCas9 to transcriptional regulators such as the Krüppel-associated box (KRAB) was found to remarkably enhance the repression efficiency [8], similar approaches to fuse ddCpf1 with regulators might be tried in human cells in the future. While this manuscript was in preparation, a paper was published to show that ddCpf1 fused to three copies of the SRDX transcriptional repressor was effective in repression of a non-coding RNA (miR159b) in plant, which was achieved by targeting the promoter region [43].

Although CRISPRa was not tested in this study, ddCpf1 in theory can be employed for activation of target genes in a similar way to that of dCas9, which could be achieved via engineering either ddCpf1 (for example, in fusion with VP64 in mammalian cells [8, 44] or with the ω-subunit of the RNAP in bacteria [7]) or the crRNA (for example, to recruit transcriptional activators via RNA aptamers [45]) to activate the target genes.

At last, it was worthy to mention that we named the Cpf1 mutant as ddCpf1 instead of dCpf1 in this study. Because Cpf1 harbors both the DNase activity and the RNase activity within distinct active domains, we here designated the DNase-dead Cpf1 as ddCpf1 to distinguish from those RNase-dead and nuclease-dead Cpf1 mutants.

## Materials and methods

### Plasmids construction

The AsCpf1 gene was cloned into pXX55-1 [46] to replace the Cas9 gene, obtaining pXX55-AsCpf1. In brief, the vector was amplified with primers of pXX55-1-zai-F and pXX55-1-zai-R, employing pXX55-1 as the template. Then, AsCpf1 was amplified from pET28a-TEV-AsCpf1 [47] using primers of pXX55-1-AsCpf1-F and pXX55-1-AsCpf1-R. With the Ezmax seamless cloning kit (Tolo Biotech., Shanghai, China), the AsCpf1 fragment and the pXX55-1 vector backbone were seamlessly assembled and verified by sequencing. To obtain a ddAsCpf1 expressing construct, pXX55-AsCpf1 was employed as the template, and primers of AsCpf1-E993A-F and AsCpf1-E993A-R were used for amplification, followed by DpnI treatment and direct transformation into DH10B competent cells. The E993A mutation in pXX55-ddAsCpf1 was confirmed by Sanger sequencing.

The Cpf1 crRNA expression vector was constructed on the basis of pgRNA-bacteria [6]. In brief, primers of BsmBI-gRNA-f and BsmBI-gRNA-r2 were used for PCR amplification of pgRNA-bacteria, followed by DpnI treatment, assembly with the Ezmax seamless assembly kit (Tolo Biotech.) and subsequent transformation into DH10B competent cells, obtaining pTC17014. Notably, one DR sequence and two BsmBI sites were introduced after the J23119-SpeI promoter, and the two BsmBI sites facilitate further insertion of different guide sequences. Meanwhile, to remove the BsmBI site from the original vector, a pair of primers (BsmBI-Muf and BsmBI-Mur) were designed and used for amplification of pTC17014, which amplicon was then treated by DpnI and Ezmax seamless assembly kit before being transformed into DH10B competent cells, generating pTC17014r. To insert one guide sequence, a pair of oligos were heat denatured and annealed, and then inserted into the BsmBI-treated pTC17014r. For multiple guide sequences, pairs of oligos were first individually annealed and then mixed together to be ligated into the BsmBI-treated pTC17014r. All crRNA sequences were verified by sequencing with primer of pcrRNA-ZF.

Plasmid pXX55-1 was firstly employed as the template and amplified with pXX55-1F and pXX55-1R, and the amplicon was then self-ligated to generate pZCas9. To construct the dCas9 expression plasmid, pZCas9 was then amplified with primers of dCas9-zai-F/dCas9-zai-R and dCas9-D10A-F/dCas9-H840A-R to produce two fragments, which were then seamlessly assembled to obtain pZX23 for expression of dCas9. Construction of the plasmid pGRNA-dlacZ to express sgRNA-NT1 targeting the NT1 site in the NT strand of *lacZ* was described before [46]. Then, pGRNA-dlacZ was used as the template and amplified with primers of pGRNA-dlacZ-1-F and pGRNA-dlacZ-1-R, whose amplicon was treated with the Ezmax seamless assembly kit (Tolo Biotech.) followed by transformation into DH10B to generate pGRNA-dlacZ(T), which expressed the sgRNA-T1 specifically targeting the T1 site in the T strand of *lacZ*.

To express ddAsCpf1 in mammalian cells, plasmid pX459-ddAsCpf1 was constructed. In brief, pXX55-1-ddAsCpf1 was used as the template for amplification of ddAsCpf1 with primers of ddAsCpf-F and ddAsCpf-R. Then, pSpCas9(BB)-2A-Puro (PX459) [48] was amplified with primers of pX459-F and pX459-R to generate the backbone. Both amplicons were purified, digested with DpnI and then seamlessly assembled with Ezmax seamless assembly kit (Tolo Biotech.) to generate pX459-ddAsCpf1. To facilitate the insertion of guide sequences into the plasmid, we first constructed pMD19T-U6-6T, containing two BspMI sites. In brief, the U6 promoter region was amplified from pSpCas9(BB)-2A-Puro (PX459) with primers of U6P-F/U6P-R1, whose amplicon was further amplified with primers of U6P-F/U6P-R2. Then, the amplified U6P fragment was inserted into the pMD19T vector (TaKaRa, Shiga, Japan), generating pMD19T-U6-6T. Paired oligos containing the DR sequence and guide sequence were heat denatured, annealed and then ligated with BspMI-treated pMD19T-U6-6T. The U6P-DR-guide fragment was then excised from correct plasmids with NcoI and XbaI digestion and then inserted into the PciI and XbaI-digested pX459-ddAsCpf1, generating the plasmids for targeted repression of genes in mammalian cells.

All primers used in this study were listed in Supplementary Table S1, and plasmids and strains could be found in Supplementary Table S2.

### Real-time reverse transcription (RT)-PCR

#### For *E. coli*.

Strains were grown in liquid LB medium with appropriate antibiotics at 37 °C overnight with shaking at 220 r.p.m., then 50 μl culture was inoculated into 5 ml fresh liquid LB medium and further incubated till the OD_600_ reached 1.2. To derepress the transcription of *lac* operon, 0.5 mm IPTG was added and cells were cultured for further 1 h before cell harvesting. To shock with nitrogen-limited conditions, cells were first washed with liquid M9 medium, and then resuspended in liquid M9 medium and cultured for another 1 h. Total RNA was extracted using the ZR Fungal/Bacterial RNA MiniPrep (Zymo Research, Irvine, CA, USA), and further treated with RNase-free DNase I (TaKaRa) to prevent contamination of trace genomic DNA.

#### For HEK293T.

Cells were grown at 37 °C under 5% CO_2_ atmosphere, in Dulbecco’s modified Eagle’s medium (Invitrogen, Carlsbad, CA, USA) supplemented with 10% heat-inactivated FCS, 100 U ml^−1^ penicillin and 100 μg ml^−1^ streptomycin. HEK293T cells were seeded into 24-well plates (Corning, Corning, NY, USA) and incubated for 20 h before being transfected with Lipofectamine 3000 transfection Reagent (Thermo Fisher Scientific, Waltham, MA, USA) at 70–80% confluency, following the manufacturer’s protocol. For each well in a 24-well plate, 500 ng plasmid DNA was used for transfection. The transfected cells were then incubated for 48 h at 37 °C under 5% CO_2_ atmosphere before being used for total RNA isolation with TRIzol Reagent (Thermo Fisher Scientific).

#### Real-time RT-PCR.

RT was performed using the PrimeScript II 1st Strand cDNA Synthesis Kit (TaKaRa), and PCR was carried out with the reaction mixture (from 100 ng RNA) as the template, using the SYBR qPCR master mix (TaKaRa) and the StepOnePlus Real-Time PCR machine (Thermo Fisher Scientific). The *gapA* gene was employed as an internal control for analysis of *E. coli* samples, while *hGAPDH* was used for mammalian cells. Three independent samples were employed for analyses.

### RNA-seq procedures and the data analysis

*E. coli* was cultivated in the same conditions as described above and 1 mm IPTG was added to relieve the repression of LacI. Then, total RNA was prepared with the RNeasy mini kit (Qiagen, Germany), and strand-specific libraries were prepared with the TruSeq Stranded Total RNA Sample Preparation kit (Illumina, San Diego, CA, USA), following the manufacturers’ procedures. Before reverse transcription, ribosomal RNA was removed using Ribo-Zero rRNA removal kit (Bacteria; Illumina, USA). The purified mRNA was first fragmented using divalent cations before being employed for library preparation, which was performed following the same procedure as described before [49]. To confirm the quality, the purified libraries were quantified by Qubit 2.0 Fluorometer (Life Technologies, Carlsbad, CA, USA) and validated with Agilent 2100 bioanalyzer (Agilent Technologies, USA). Cluster was generated by cBot with the library diluted to 10 pm, which were then sequenced on the Illumina HiSeq 2500 (Illumina, USA) by Shanghai Biotechnology Corporation.

Raw reads were first preprocessed to filter out the low-quality reads, the rRNA reads, the sequencing adapters and those short-fragment reads. Tophat v2.1.0 [50] was used to map the processed reads to the *E. coli* K-12 MG1655 reference genome with two mismatches. After that, Cufflinks v2.1.1 [51] was employed to generate FPKM (fragments per kilobase million) values for known gene models, and those differentially expressed genes were identified using Cuffdiff [51] with the *P*-value significance threshold set with the false discovery rate [52]. Fold-changes were also calculated based on the FPKM values.

### Phenotype analysis

*E. coli* strains were cultured overnight in liquid LB medium with appropriate antibiotics and then diluted with sterile water to reach OD_600_=2.0. Then, cells were serially diluted with sterile water before being dotted on plates. Plates were then cultured at 37 °C overnight to observe the growth phenotypes. To validate bacterial growth in nitrogen-limited conditions, M9 medium was used and M9 with glutamine supplementation was employed as a control. And when cultured on M9 medium, a longer incubation time (for example, up to 36 h) might be needed.
