# Supplementary material for: Multiplex gene regulation by CRISPR-ddCpf1
Source: Cell Discov. 2017 Jun 6;3:17018–. doi: 10.1038/celldisc.2017.18 (PMC5460296; doi:10.1038/celldisc.2017.18)
Supplement: Supplementary Information [file celldisc201718-s1.pdf]

# Supplementary Information

## Supplementary Tables

**Table S1. Oligos used in this study**

| **Name** | **Sequence (5'-3')** | **Description** |
|---|---|---|
| **Oligos for plasmids construction** | | |
| pXX55-1-zai-R | ctagtatttctcctctttctctagt | amplification of the vector pXX55-1 |
| pXX55-1-zai-F | tactagcaggccgtgccggcacg | |
| pXX55-1-AsCpf1-F | actagagaaagaggagaaatactagatgacacagttcgagggctttacc | amplification of AsCpf1 gene |
| pXX55-1-AsCpf1-R | acgtgccggcacggcctgctagtactagttcctcagctcctggatgtag | |
| AsCpf1-E993A-F | tcaggttggccagcaccaccacggcctggtag | mutation of E993 to alanine |
| AsCpf1-E993A-R | ggtgctggccaacctgaatttcggctttaagag | |
| BsmBI-gRNA-f | gagacgacctgcccgtctcgttttttgaagcttgggcccgaac | insertion of both DR and two BsmBI sites after the crRNA promoter |
| BsmBI-gRNA-r2 | gacgggcaggtcgtctcgatctacaagagtagaaattactagtattatacctaggactg | |
| BsmBI-Muf | caaggtctcattttcgccaaaag | mutation of the BsmBI site on the backbone of the crRNA plasmid |
| BsmBI-mur | ggcgaaaatgagaccttgatcg | |
| pXX55-1F | accagtagaaacagacgaagaatcc | to generate plasmid pZCas9 |
| pXX55-1R | gccgtcaagttgtcataataaatcaca | |
| dCas9-zai-F | cgtttaagtgattatgatgtcgatgccattgttccacaaagtttcct | amplification of pZCas9 backbone, to construct pZX23 |
| dCas9-zai-R | atccgacgctatttgtgccgatggctaagcctattgagtatttcttatcc | |
| dCas9-D10A-F | agccatcggcacaaatagcgtcggat | introduction of two mutated sites into Cas9 to generate dCas9 |
| dCas9-H840A-R | ggcatcgacatcataatcacttaaacg | |
| pGRNA-dlacZ-1-F | tcgttttacaacgtcgtgacgttttagagctagaaatagc | construction of pGRNA-dlacZ(T) |
| pGRNA-dlacZ-1-R | gtcacgacgttgtaaaacgaactagtattatacctaggac | |
| pcrRNA-ZF | gtatcaacagggacaccag | crRNA sequencing primer |
| lacZcrRNA-T1F | agatcaacgtcgtgactgggaaaaccc | cloning the *lacZ* T1 guide, targeting the T strand |
| lacZcrRNA-T1R | aaaagggttttcccagtcacgacgttg | |
| lacZcrRNA-T2F | agatgccagctggcgtaatagcgaaga | cloning the *lacZ* T2 guide, targeting the T strand |
| lacZcrRNA-T2R | aaaatcttcgctattacgccagctggc | |
| lacZcrRNA-T3F | agatatgttgatgaaagctggctacag | cloning the *lacZ* T3 guide, targeting the T strand |
| lacZcrRNA-T3R | aaaactgtagccagctttcatcaacat | |
| lacZcrRNA-T4F | agattcgtgactgggaaaaccctggcg | cloning the *lacZ* T4 guide, targeting the T strand, without a PAM site |
| lacZcrRNA-T4R | aaaacgccagggttttcccagtcacga | |
| lacZcrRNA-NT1F | agatccagtcacgacgttgtaaaacga | cloning the *lacZ* NT1 guide, targeting the NT strand |
| lacZcrRNA-NT1R | aaaatcgttttacaacgtcgtgactgg | |
| lacZcrRNA-NT2F | agatcggcaccgcttctggtgccggaa | cloning the *lacZ* NT2 guide, targeting the NT strand |
| lacZcrRNA-NT2R | aaaattccggcaccagaagcggtgccg | |

| | | |
|---|---|---|
| lacZcrRNA-NT3F | agataggggacgacgacagtatcggcc | cloning the *lacZ* NT3 guide, targeting the NT strand |
| lacZcrRNA-NT3R | aaaaggccgatactgtcgtcgtcccct | |
| malTcrRNA-TF | agatcacagtgaagtgattaactatgc | cloning the *malT* T guide, targeting the T strand |
| malTcrRNA-TR | aaaaagcatagttaatcacttcactgtg | |
| proPcrRNA-TF | agatttgcttacgcattaggtaaagtt | cloning the *proP* T guide, targeting the T strand |
| proPcrRNA-TR | aaaaaactttacctaatgcgtaagcaa | |
| degPcrRNA-TF | agatgcgttatctccgctctctgcaac | cloning the *degP* T guide, targeting the T strand |
| degPcrRNA-TR | aaaagttgcagagagcggagataacgc | |
| rseAcrRNA-TF | agatatggatggcgaaacgctggatag | cloning the *rseA* T guide, targeting the T strand |
| rseAcrRNA-TR | aaaactatccagcgtttcgccatccat | |
| malTPcrRNA-NTF | agatcgcttcgtaattaatggttataa | cloning the *malT* P guide, targeting the NT strand in the promoter region |
| malTPcrRNA-NTR | aaaattataaccattaattacgaagcg | |
| malTPcrRNA-TF | agattggccgaccttataaccattaat | cloning the *malT* P guide, targeting the T strand in the promoter region |
| malTPcrRNA-TR | aaaaattaatggttataaggtcggcca | |
| glnGcrRNA-TF | agattggcaacaggcacgcagcccgat | cloning the *glnG* guide, targeting the T strand in the promoter region |
| glnGcrRNA-TR | aaaaatcgggctgcgtgcctgttgcca | |
| cpxRcrRNA-TF | agattgcctcggaggtatttaaacaat | cloning the *cpxR* guide, targeting the T strand in the promoter region |
| cpxRcrRNA-TR | aaaaattgtttaaatacctccgaggca | |
| arcAcrRNA-TF | agatggtagcaaacatgcagaccccgc | cloning the *arcA* guide, targeting the T strand in the promoter region |
| arcAcrRNA-TR | aaaagcggggtctgcatgtttgctacc | |
| ddAsCpf-F | tcggtatccacggagtcccagcagccatgacacagttcgagggctttac | amplification of the AsCpf1 gene |
| ddAsCpf-R | ctttttcgtggccgccggccttttgttcctcagctcctggatgtaggc | |
| pX459-F | aaaaggccggcggccacgaaaaaggc | amplification of pX459 backbone |
| pX459-R | ggctgctgggactccgtggataccgaccttc | |
| U6P-F | ccatggacatgtgagggcctatttcccatg | construction of pMD19T-U6-6T |
| U6P-R1 | ggatccacctgcaattcggtgtttcgtcctttccac | |
| U6P-R2 | ggtacctctagaaaaaaatatcgcaggtggatccacctgcaattcggtg | |
| DNMT1-crRNA-P1F | accgaatttctactcttgtagatagacaaggtcttgctctgtctcc | cloning the DR-P1 sequence, targeting P1 site the *DNMT1* gene |
| DNMT1-crRNA-P1R | aaaaggagacagagcaagaccttgtctatctacaagagtagaaatt | |
| DNMT1-crRNA-P2F | accgaatttctactcttgtagattccccatcacacctgaaagaatg | cloning the DR-P2 sequence, targeting P1 site the *DNMT1* gene |
| DNMT1-crRNA-P2R | aaaacattctttcaggtgtgatggggaatctacaagagtagaaatt | |
| DNMT1-crRNA-P3F | accgaatttctactcttgtagatgaccagcctaggtaacatagaga | cloning the DR-P3 sequence, targeting P1 site the *DNMT1* gene |
| DNMT1-crRNA-P3R | aaaatctctatgttacctaggctggtcatctacaagagtagaaatt | |
| DNMT1-crRNA-P4F | accgaatttctactcttgtagatctgggaggtgggcacggtgcccg | |

| | | |
|---|---|---|
| DNMT1-crRNA-P4R | aaaacgggcaccgtgcccacctcccagatctacaagagtagaaatt | cloning the DR-P4 sequence, targeting P1 site the *DNMT1* gene |
| DNMT1-crRNA-T1F | accgaatttctactcttgtagatgcgccaaaatggaccgtggattc | cloning the DR-T1 sequence, targeting P1 site the *DNMT1* gene |
| DNMT1-crRNA-T1R | aaaagaatccacggtccattttggcgcatctacaagagtagaaatt | |
| DNMT1-crRNA-T2F | accgaatttctactcttgtagatgaaagagacagcttaacagaaaa | cloning the DR-T2 sequence, targeting P1 site the *DNMT1* gene |
| DNMT1-crRNA-T2R | aaaattttctgttaagctgtctctttcatctacaagagtagaaatt | |
| DNMT1-crRNA-NT1F | accgaatttctactcttgtagatgcagtacccacattcccaagcta | cloning the DR-NT1 sequence, targeting P1 site the *DNMT1* gene |
| DNMT1-crRNA-NT1R | aaaatagcttgggaatgtgggtactgcatctacaagagtagaaatt | |
| DNMT1-crRNA-NT2F | accgaatttctactcttgtagatgccaggtagccctcctacagcag | cloning the DR-NT2 sequence, targeting P1 site the *DNMT1* gene |
| DNMT1-crRNA-NT2R | aaaactgctgtaggagggctacctggcatctacaagagtagaaatt | |
| **Oligos for assembly of arrays** | | |
| arrayG1-f1 | agatagccggtcgaagcggaagattataatttctactcttgtagatc | assembly of array G1 |
| arrayG1-f2 | atgccgttcagatgatcctctcaatttctactcttgtagatgccg | |
| arrayG1-f3 | acaacgggctgaatggctaaatttctactcttgtagataagccggggc | |
| arrayG1-f4 | taatcttgctcgaaatttctactcttgtagatatgaggatatgtgacaaa | |
| arrayG1-f5 | cgttcaatttctactcttgtagatattctgctggatatcaacttacc | |
| arrayG1-r1 | gcatgatctacaagagtagaaattataatcttccgcttcgaccggct | |
| arrayG1-r2 | ttgtcggcatctacaagagtagaaattgagaggatcatctgaacg | |
| arrayG1-r3 | attagccccggcttatctacaagagtagaaatttagccattcagcccg | |
| arrayG1-r4 | aacgtttgtcacatatcctcatatctacaagagtagaaatttcgagcaag | |
| arrayG1-r5 | aaaaggtaagttgatatccagcagaatatctacaagagtagaaattg | |
| arrayG2-f1 | agatactgccgattctggtattaaccgaatttctactcttgtagata | assembly of array G2 |
| arrayG2-f2 | cagacgtccaaggagatacccaaatttctactcttgtagatgctc | |
| arrayG2-f3 | gcttctggattcatggtttaatttctactcttgtagattggaagatga | |
| arrayG2-f4 | tgcggaagtcggtaatttctactcttgtagatggttgtggatgactttt | |
| arrayG2-f5 | ccaccaaatttctactcttgtagatcgtccaatgcccgttgtgatggt | |
| arrayG2-r1 | tctgtatctacaagagtagaaattcggttaataccagaatcggcagt | |
| arrayG2-r2 | aagcgagcatctacaagagtagaaatttgggtatctccttggacg | |
| arrayG2-r3 | cgcatcatcttccaatctacaagagtagaaattaaaccatgaatccag | |
| arrayG2-r4 | gtggaaaagtcatccacaaccatctacaagagtagaaattaccgacttc | |
| arrayG2-r5 | aaaaaccatcacaacgggcattggacgatctacaagagtagaaatttg | |
| arrayG3-f1 | agatatcaacgttctacttgttgatgaaatttctactcttgtagatcg | assembly of array G3 |
| arrayG3-f2 | aagcgggttatgtcatcgatgaatttctactcttgtagatcagaga | |
| arrayG3-f3 | gaagtatgaccgagttaaatttctactcttgtagatgcgatgactat | |
| arrayG3-f4 | ggaataagcgtcaatttctactcttgtagataggtgatgattgtggatg | |

| | | |
|---|---|---|
| arrayG3-f5 | atcataatttctactcttgtagataagactctacagcactgatcaac | |
| arrayG3-r1 | gcttcgatctacaagagtagaaatttcatcaacaagtagaacgttgat | |
| arrayG3-r2 | cttctctctgatctacaagagtagaaattcatcgatgacataaccc | |
| arrayG3-r3 | ttccatagtcatcgcatctacaagagtagaaatttaactcggtcata | |
| arrayG3-r4 | tgatcatccacaatcatcacctatctacaagagtagaaattgacgctta | |
| arrayG3-r5 | aaaagttgatcagtgctgtagagtcttatctacaagagtagaaatta | |
| arrayG4-f1 | agataccgatcaacccgcagggaaatcaatttctactcttgtagatcg | assembly of array G4 |
| arrayG4-f2 | ggaaacattgtgctgatgctgaatttctactcttgtagattcatccgt | |
| arrayG4-f3 | atccgcctgtttcgcaatttctactcttgtagatttattggtcgatg | |
| arrayG4-f4 | acgatccgggaatttctactcttgtagatctgatagaagatgacat | |
| arrayG4-f5 | gctgataatttctactcttgtagatggagtacaaacaatgcaagagaa | |
| arrayG4-r1 | ttcccgatctacaagagtagaaattgatttccctgcgggttgatcggt | |
| arrayG4-r2 | ggatacggatgaatctacaagagtagaaattcagcatcagcacaatgt | |
| arrayG4-r3 | tcgtcatcgaccaataaatctacaagagtagaaattgcgaaacaggc | |
| arrayG4-r4 | cagcatgtcatcttctatcagatctacaagagtagaaattcccgga | |
| arrayG4-r5 | aaaattctcttgcattgtttgtactccatctacaagagtagaaattat | |
| arrayG5-f1 | agatcccgccaggacaagaccatgatcaatttctactcttgtagattggca | assembly of array G5 |
| arrayG5-f2 | acaggcacgcagcccgataatttctactcttgtagattgcctcggaggtattt | |
| arrayG5-f3 | aaacaataatttctactcttgtagattgcaacgttctaaagactcctta | |
| arrayG5-r1 | ctgttgccaatctacaagagtagaaattgatcatggtcttgtcctggcggg | |
| arrayG5-r2 | gtttaaatacctccgaggcaatctacaagagtagaaattatcgggctgcgtgc | |
| arrayG5-r3 | aaaataaggagtctttagaacgttgcaatctacaagagtagaaattatt | |
| arrayG6-f1 | agattgcgccgaccaatatcgctgcgcaatttctactcttgtagatttgtc | assembly of array G6 |
| arrayG6-f2 | gttaccgatatgcaaagtaatttctactcttgtagattgcaacgggaaacg | |
| arrayG6-f3 | gtctggttaaatttctactcttgtagatggtagcaaacatgcagaccccgc | |
| arrayG6-r1 | taacgacaaatctacaagagtagaaattgcgcagcgatattggtcggcgca | |
| arrayG6-r2 | agaccgtttcccgttgcaatctacaagagtagaaattactttgcatatcgg | |
| arrayG6-r3 | aaaagcggggtctgcatgtttgctaccatctacaagagtagaaatttaacc | |
| **Oligos for realtime RT-PCR assays** | | |
| gapA-qF | caacgacctgttagacgctgatt | |
| gapA-qR | acgttcagcggtaacacggatt | |
| lacZ-qF | gacgtctcgttgctgcataaac | |
| lacZ-qR | tcgccgcacatctgaacttca | |
| rpoE-qF | acatggctgtatcggattgc | |
| rpoE-qR | aatggcatccacatcactgg | |
| malT-qF | ctctaacgccaacaaagtccg | |
| malT-qR | tagccgtatgacgcaaccag | |
| proP-qF | cgatgttcccgacgcatatc | |
| proP-qR | catcaggtaataggcaggcatc | |
| degP-qF | aggtgatgccttcagtggtc | |

| | | |
|---|---|---|
| degP-qR | gagctctggaacggagaacc | |
| rseA-qF | tacgtcaaccggcgacattg | |
| rseA-qR | acgcccatttgggtaagctg | |
| glnG-qF | agggatagtctgggtagtcg | |
| glnG-qR | gttctcaaacgtcgtacagg | |
| cpxR-qF | ttgagctgggcgcagatgac | |
| cpxR-qR | tttgctgttgctcgctccag | |
| DNMT1-qF | gagccaaatcggatgagtc | |
| DNMT1-qR | aggaagcggtctagcaac | |
| hGAPDH-qF | gagtcaacggatttggtcgt | |
| hGAPDH-qR | gacaagcttcccgttctcag | |

## Table S2. Strains and plasmids used in this study

| **Name** | **Relevant properties or genotypes** | **Sources** |
|---|---|---|
| ***E. coli* strains** | | |
| MG1655 | F- lambda- *ilvG- rfb*-50 *rph*-1 | Lab stock |
| *E. coli* DH10B | $F^-$ *endA1 deoR*$^+$ *recA1 galE15 galK16 nupG rpsL* Δ*(lac)X74* φ80*lacZΔM15 araD139* Δ*(ara,leu)7697 mcrA* Δ*(mrr-hsdRMS-mcrBC)* $Str^R$ $\lambda^-$ | Invitrogen |
| **Plasmids** | | |
| pXX55-AsCpf1 | pXX55-1 with *AsCpf1* gene replacing the original *cas9* gene | This study |
| pXX55-ddAsCpf1 | Constructed on the basis of pXX55-AsCpf1, with E993A mutation on AsCpf1 | This study |
| pTC17014r | Constructed on the basis of pgRNA-bacteria [1], with a DR sequence and two BsmBI sites following the J23119-SpeI promoter, allowing for easy insertion of guide sequences | This study |
| pTC160300 | pTC17014r carrying the guide sequence targeting the NT1 site on *lacZ* gene | This study |
| pTC160301 | pTC17014r carrying the guide sequence targeting the NT2 site on *lacZ* gene | This study |
| pTC160302 | pTC17014r carrying the guide sequence targeting the NT3 site on *lacZ* gene | This study |
| pTC160303 | pTC17014r carrying the guide sequence targeting the T2 site on *lacZ* gene | This study |
| pTC160304 | pTC17014r carrying the guide sequence targeting the T3 site on *lacZ* gene | This study |
| pTC160305 | pTC17014r carrying the guide sequence targeting the T1 site on *lacZ* gene | This study |
| pTC160309 | pTC17014r carrying the guide sequence targeting the T strand on *malT* gene | This study |
| pTC160310 | pTC17014r carrying the guide sequence targeting the T strand in *lacZ* promoter | This study |

| | | |
|---|---|---|
| pTC160311 | pTC17014r carrying the guide sequence targeting the T strand in *malT* promoter | This study |
| pTC160312 | pTC17014r carrying the guide sequence targeting the NT strand in *malT* promoter | This study |
| pTC17015 | pTC17014r carrying the guide sequence targeting the NT strand in *ftsZ* promoter | This study |
| pTC17016 | pTC17014r carrying the guide sequence targeting the T1 site on *ftsZ* gene (T strand) | This study |
| pTC17017 | pTC17014r carrying the guide sequence targeting the T2 site on *ftsZ* gene (T strand) | This study |
| pTC17041 | pTC17014r carrying the guide sequence targeting the T strand on *degP* gene | This study |
| pTC17043 | pTC17014r carrying the guide sequence targeting the T strand on *rseA* gene | This study |
| pTC17046 | pTC17014r carrying the guide sequence targeting the T strand on *basS* gene | This study |
| pTC17050 | pTC17014r carrying the guide sequence targeting the T strand on *proP* gene | This study |
| pTC17055 | pTC17014r carrying the guide sequence targeting the T strands of *malT*, *proP*, *degP* and *rseA* | This study |
| pTC17056 | pTC17014r carrying the guide sequence targeting the T strands of *proP*, *degP*, *rseA* and *malT* | This study |
| pTC17089 | pTC17014r carrying the guide sequence targeting the T strand on *uhpA* gene | This study |
| pTC17090 | pTC17014r carrying the guide sequence targeting the T strand on *glnG* gene | This study |
| pTC17091 | pTC17014r carrying the guide sequence targeting the T strand on *cpxR* gene | This study |
| pTC17092 | pTC17014r carrying the guide sequence targeting the T strand on *zraR* gene | This study |
| pTC17093 | pTC17014r carrying the guide sequence targeting the T strand on *dcuR* gene | This study |
| pTC17094 | pTC17014r carrying the guide sequence targeting the T strand on *creB* gene | This study |
| pTC17095 | pTC17014r carrying the guide sequence targeting the T strand on *arcA* gene | This study |
| pTC17099 | pTC17014r carrying the guide sequence targeting the T strands of array G1 (6 genes, including *phoB*, *sfmZ*, *cusR*, *citB*, *kdpE* and *torR*) | This study |
| pTC17100 | pTC17014r carrying the guide sequence targeting the T strands of array G2 (6 genes, including *phoP*, *narL*, *rssB*, *rstA*, *cheY* and *cheB*) | This study |
| pTC17101 | pTC17014r carrying the guide sequence targeting the T strands of array G3 (6 genes, including *uvrY*, *yedW*, *baeR*, *yehT*, *narP* and *rcsB*) | This study |

| | | |
|---|---|---|
| pTC17102 | pTC17014r carrying the guide sequence targeting the T strands of array G4 (6 genes, including *atoC*, *evgA*, *ypdB*, *glrR*, *qseB* and *ompR*) | This study |
| pTC17103 | pTC17014r carrying the guide sequence targeting the T strands of array G5 (4 genes, including *uhpA*, *glnG*, *cpxR* and *zraR*) | This study |
| pTC17104 | pTC17014r carrying the guide sequence targeting the T strands of array G6 (4 genes, including *basS*, *dcuR*, *creB* and *arcA*) | This study |
| pZCas9 | A plasmid carrying Cas9 gene, generated from pXX55-1 | This study |
| pZX23 | A plasmid carrying dCas9 gene, generated from pZCas9 (p15A ori) ($Kan^r$) | This study |
| pGRNA-dlacZ | A plasmid expressing an sgRNA specifically targeting the NT1 site in *lacZ* | [2] |
| pGRNA-dlacZ(T) | A plasmid expressing an sgRNA specifically targeting the T1 site in *lacZ* | This study |
| pX459-ddAsCpf1 | Constructed on the basis of pSpCas9(BB)-2A-Puro (PX459) [3], with SpCas9 replaced by ddAsCpf1 | This study |
| pMD19T-U6-6T | The pMD19T vector inserted with U6 promoter, followed by two BspMI sites | This study |
| pX459-ddAsCpf1-DNMT-P1 | Constructed on the basis of pX459-ddAsCpf1, expressing both ddAsCpf1 and crRNA targeting to the P1 site in the promoter region of DNMT1 | This study |
| pX459-ddAsCpf1-DNMT-P2 | Constructed on the basis of pX459-ddAsCpf1, expressing both ddAsCpf1 and crRNA targeting to the P2 site in the promoter region of DNMT1 | This study |
| pX459-ddAsCpf1-DNMT-P3 | Constructed on the basis of pX459-ddAsCpf1, expressing both ddAsCpf1 and crRNA targeting to the P3 site in the promoter region of DNMT1 | This study |
| pX459-ddAsCpf1-DNMT-P4 | Constructed on the basis of pX459-ddAsCpf1, expressing both ddAsCpf1 and crRNA targeting to the P4 site in the promoter region of DNMT1 | This study |
| pX459-ddAsCpf1-DNMT-T1 | Constructed on the basis of pX459-ddAsCpf1, expressing both ddAsCpf1 and crRNA targeting to the T1 site in the coding region of DNMT1 | This study |
| pX459-ddAsCpf1-DNMT-T2 | Constructed on the basis of pX459-ddAsCpf1, expressing both ddAsCpf1 and crRNA targeting to the T2 site in the coding region of DNMT1 | This study |
| pX459-ddAsCpf1-DNMT-NT1 | Constructed on the basis of pX459-ddAsCpf1, expressing both ddAsCpf1 and crRNA targeting to the NT1 site in the coding region of DNMT1 | This study |
| pX459-ddAsCpf1-DNMT-NT2 | Constructed on the basis of pX459-ddAsCpf1, expressing both ddAsCpf1 and crRNA targeting to the NT2 site in the coding region of DNMT1 | This study |

**Table S3. Grouping of the 32 *E. coli* response regulators tested in this study***

| | | | | | | |
|---|---|---|---|---|---|---|
| array-G1 | *phoB* (b0399) | *sfmZ* (b0535) | *cusR* (b0571) | *citB* (b0620) | *kdpE* (b0694) | *torR* (b0995) |

| | | | | | | |
|---|---|---|---|---|---|---|
| array-G2 | *phoP* (b1130) | *narL* (b1221) | *rssB* (b1235) | *rstA* (b1608) | *cheY* (b1882) | *cheB* (b1883) |
| array-G3 | *uvrY* (b1914) | *yedW* (b1969) | *baeR* (b2079) | *yehT*# (b2125) | *narP* (b2193) | *rcsB* (b2217) |
| array-G4 | *atoC* (b2220) | *evgA* (b2369) | *ypdB*# (b2381) | *glrR* (b2554) | *qseB* (b3025) | *ompR* (b3405) |
| array-G5 | *uhpA* (b3669) | *glnG*# (b3868) | *cpxR* (b3912) | *zraR*# (b4004) | / | / |
| array-G6 | *basR*# (b4112) | *dcuR*# (b4124) | *creB* (b4398) | *arcA* (b4401) | / | / |

*, Both gene names and the "b number" names were provided for the response regulators, and genes were sorted in the "b" numerical order.

#, As the kinase-encoding genes and the regulator-encoding genes co-transcribed, the kinase genes, which are in front of the regulator genes, were selected as the targets in these assays, resulting in the silencing of both kinase and regulator.

**Table S4. Sequences of the customized crRNA arrays used in this study***

| |
|---|
| >array G1 sequence:<br>AATTTCTACTCTTGTAGATagccggtcgaagcggaagattatAATTTCTACTCTTGTAGATcatgccgttcagatgatcctctcAATTTCTACTCTTGTAGATgccgacaacgggctgaatggctaAATTTCTACTCTTGTAGATaagccggggctaatcttgctcgaAATTTCTACTCTTGTAGATatgaggatatgtgacaaacgttcAATTTCTACTCTTGTAGATattctgctggatatcaacttacc |
| >array G2 sequence:<br>AATTTCTACTCTTGTAGATactgccgattctggtattaaccgAATTTCTACTCTTGTAGATacagacgtccaaggagatacccaAATTTCTACTCTTGTAGATgctcgcttctggattcatggtttAATTTCTACTCTTGTAGATtggaagatgatgcggaagtcggtAATTTCTACTCTTGTAGATggttgtggatgacttttccaccaAATTTCTACTCTTGTAGATcgtccaatgcccgttgtgatggt |
| >array G3 sequence:<br>AATTTCTACTCTTGTAGATatcaacgttctacttgttgatgaAATTTCTACTCTTGTAGATcgaagcgggttatgtcatcgatgAATTTCTACTCTTGTAGATcagagagaagtatgaccgagttaAATTTCTACTCTTGTAGATgcgatgactatggaataagcgtcAATTTCTACTCTTGTAGATaggtgatgattgtggatgatcatAATTTCTACTCTTGTAGATaagactctacagcactgatcaac |
| >array G4 sequence: |

| |
|---|
| AATTTCTACTCTTGTAGATaccgatcaacccgcagggaaatcAATTTCTACTCTTGTAGATcgggaaacattgtgctgatgctgAATTTCTACTCTTGTAGATtcatccgtatccgcctgtttcgcAATTTCTACTCTTGTAGATttattggtcgatgacgatccgggAATTTCTACTCTTGTAGATctgatagaagatgacatgctgatAATTTCTACTCTTGTAGATggagtacaaacaatgcaagagaa |
| >array G5 sequence:<br>AATTTCTACTCTTGTAGATcccgccaggacaagaccatgatcAATTTCTACTCTTGTAGATtggcaacaggcacgcagcccgatAATTTCTACTCTTGTAGATtgcctcggaggtatttaaacaatAATTTCTACTCTTGTAGATtgcaacgttctaaagactcctta |
| >array G6 sequence:<br>AATTTCTACTCTTGTAGATtgcgccgaccaatatcgctgcgcAATTTCTACTCTTGTAGATttgtcgttaccgatatgcaaagtAATTTCTACTCTTGTAGATtgcaacgggaaacggtctggttaAATTTCTACTCTTGTAGATggtagcaaacatgcagaccccgc |

*, Sequence was shown from 5’ to 3’ with DR sequence in capital.

## Supplementary Figures

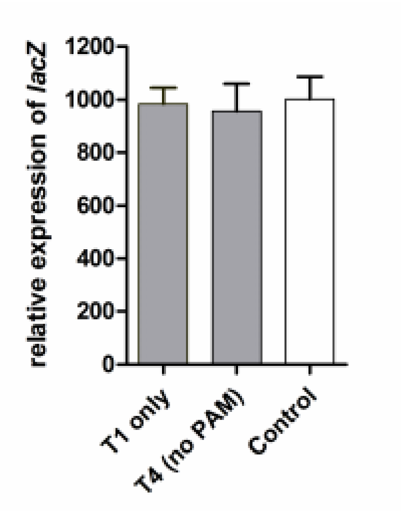


**Figure S1. Repression of *lacZ* transcription in MG1655.** Either the expression of lacZ-crRNA(T1) only or coexpression of ddCpf1 and lacZ-crRNA(T4) without a PAM site showed no significant repression of *lacZ* transcription. Most of T1 and T4 guide sequences were overlapping, while there was no PAM site nearby the T4 protospacer sequence in the template strand of *lacZ*. MG1655 expressing ddCpf1 only was employed as a control, and its *lacZ* transcriptional value was normalized to 1000.

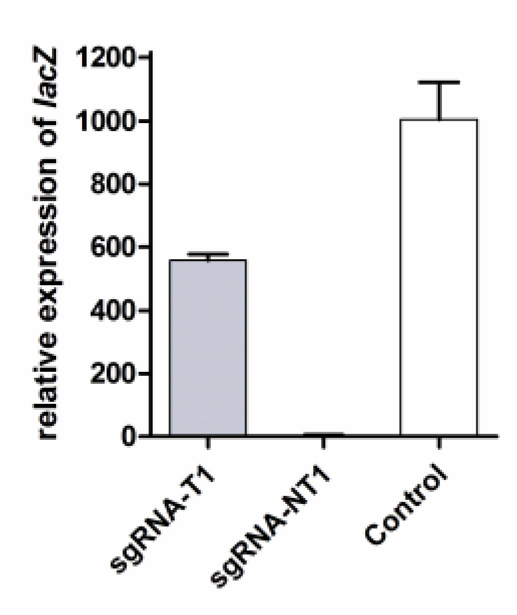


**Figure S2. Repression of *lacZ* transcription in MG1655 through coexpression of dCas9 and sgRNAs.** Positions of the guide sequences were labeled in Figure 1A and the sequences could be found in Table S1. The transcriptional level of *lacZ* in cells expressing dCas9 only was employed as a control, and its transcriptional value was normalized to 1000.

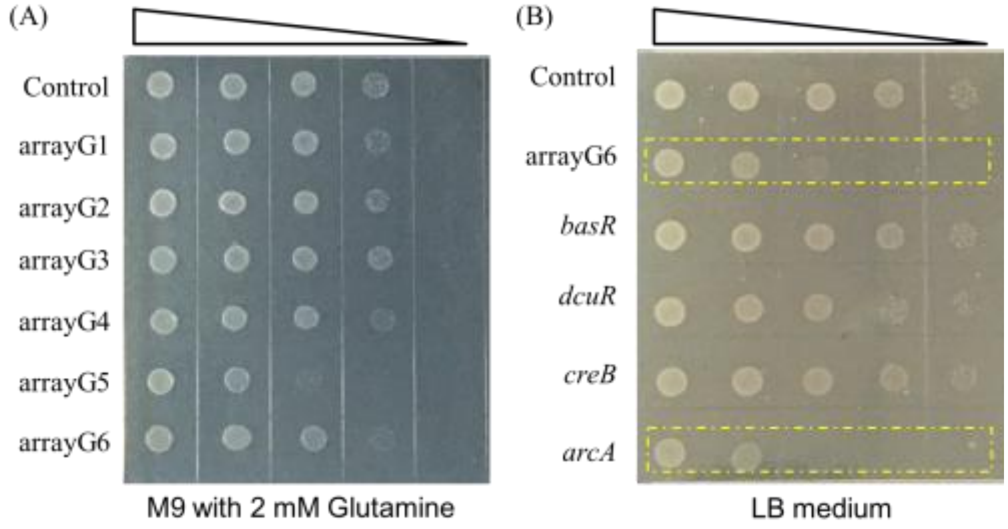


**Figure S3. Growth phenotypes of ddCpf1-mediated prompt screening of TCSs in *E. coli*.** (A) The growth phenotypes of arrayG1 to arrayG6 on M9 plate supplemented with 2 mM Glutamine. (B) ddCpf1-mediated silencing of individual genes in group 6. As indicated by yellow dashed box, the repression of *arcA* led to impaired growth on LB plate. Cells expressing ddCpf1 only were employed as the positive control.

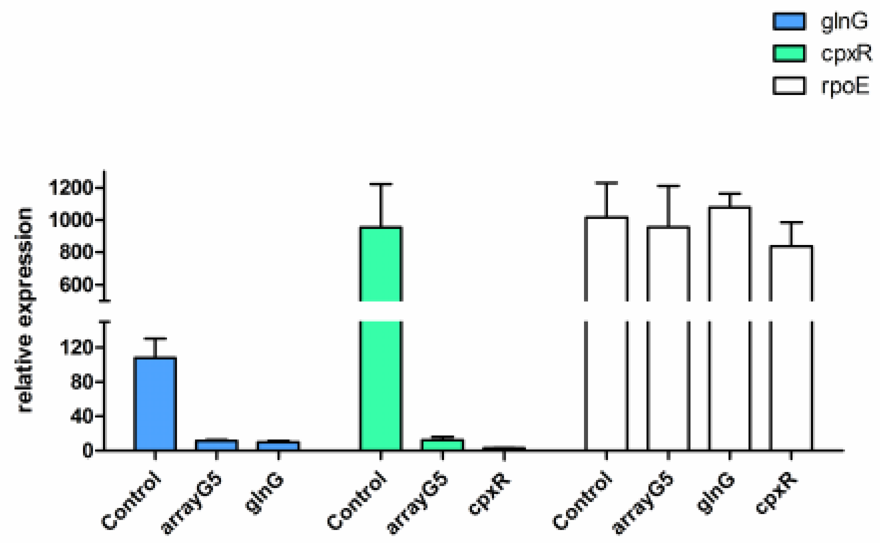


**Figure S4. Analysis of the transcription of TCSs involved in ammonium assimilation in MG1655.** Cells expressing ddCpf1 only were employed as the control. The transcription of *glnG* and *cpxR* was remarkably repressed in cells expressing ddCpf1 with either arrayG5 or individual crRNAs. The transcriptional level of the non-target *rpoE* gene in the control was normalized to 1000. arrayG5, cells expressing both ddCpf1 and arrayG5; glnG, cells expressing ddCpf1 and an individual crRNA targeting to *glnG*; cpxR, cells expressing ddCpf1 and an individual crRNA targeting to *cpxR*. No significant changes were observed for the transcriptional level of *rpoE* in each sample.

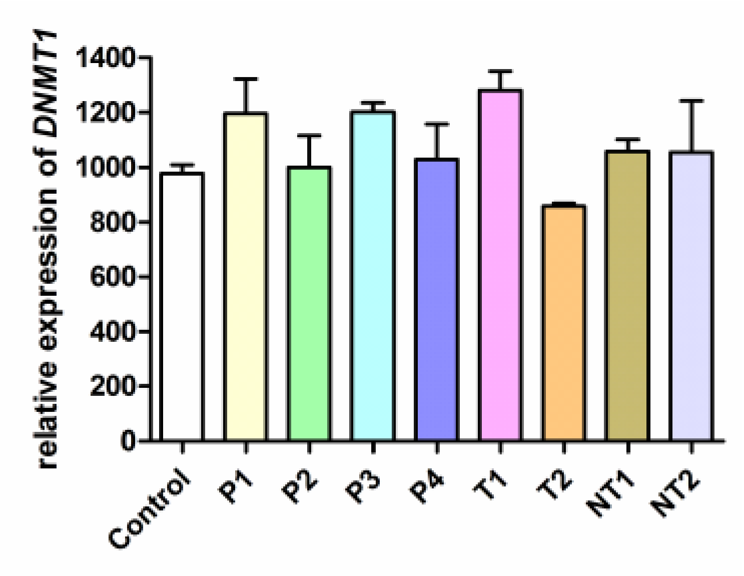


**Figure S5. Repression of *DNMT1* transcription in human HEK293T through coexpression of ddCpf1 and crRNAs.** The guide sequences of the crRNAs tested could be found in Table S1, and the transcriptional level of *DNMT1* in cells expressing ddCpf1 and a crRNA blank plasmid was employed as a control, whose transcriptional value was normalized to 1000.

1. Qi, L.S., et al., *Repurposing CRISPR as an RNA-guided platform for sequence-specific control of gene expression.* Cell, 2013. **152**(5): p. 1173-83.
2. Zheng, X., et al., *An efficient system for deletion of large DNA fragments in Escherichia coli via introduction of both Cas9 and the non-homologous end joining system from Mycobacterium smegmatis.* Biochem Biophys Res Commun, 2017. **485**(4): p. 768-774.
3. Ran, F.A., et al., *Genome engineering using the CRISPR-Cas9 system.* Nat Protoc, 2013. **8**(11): p. 2281-308.
